# Comparison of Quality of Life in Transfemoral Amputee Using Bone‐Anchored Prostheses vs. Socket Prostheses: A Systemic Review and Meta‐Analysis

**DOI:** 10.1111/os.70086

**Published:** 2025-07-18

**Authors:** Janice Tan, Nafisa Zilani, Rezaul Karim, Bijendra Patel

**Affiliations:** ^1^ Barts Cancer Institute Queen Mary University of London London UK; ^2^ Barts and the London School of Medicine and Dentistry Queen Mary University of London London UK; ^3^ GKT School of Medical Education King's College University of London London UK; ^4^ Department of Laparoscopic Surgery and Surgical Skills, Barts Cancer Institute Queen Mary University of London London UK

**Keywords:** bone‐anchored, mobility, quality of life, socket‐suspended, transfemoral amputation

## Abstract

Amputation has a profound impact on an individual's quality of life (QoL) and functional ability. While socket prostheses are the current first‐line treatment, they often cause socket‐related issues. Bone‐anchored prostheses (BAP) have been introduced to address these problems and improve the amputee experience. This systematic review and meta‐analysis aim to compare the QoL between bone‐anchored and socket prostheses in transfemoral amputees. A systematic review and meta‐analysis were conducted from November 2023 to July 2024, following PRISMA guidelines. Databases including PUBMED, EMBASE, Scopus, Cochrane, and Web of Science were searched. Studies of single‐arm trial design comparing pre‐ and post‐operative outcomes were selected based on specific inclusion and exclusion criteria. Statistical analysis was performed using inverse variance with a random effect model. The primary outcome was QoL, measured using the Questionnaire for Persons with a Transfemoral Amputation (Q‐TFA) and 36‐Item Short Form Survey (SF‐36), and the secondary outcome was mobility, assessed by the 6‐Minute Walk Test (6MWT). Subgroup analyses compared different types of BAP (Press‐fit vs. Screw‐type) on QoL. Thirteen NRCTs with 398 participants were included. Significant improvements in QoL were observed in all Q‐TFA domains and the SF‐36 Physical Component Score (PCS), but not in the SF‐36 Mental Component Score (MCS). Mobility improved significantly as measured by the six‐minute walk test (6MWT). No significant differences in QoL were found between Press‐fit and Screw‐type BAP implants. Overall, BAP significantly improve both QoL and mobility, but study limitations currently restrict their use to individuals with socket‐related complications. As such, it cannot yet be universally recommended as a first‐line intervention.

## Graphical abstract

The standard treatment for lower limb amputation is socket prostheses. This systematic review and meta‐analysis showed significant quality of life improvements with bone‐anchored prostheses (BAP) over socket prostheses. The positive findings suggest BAP has the potential to replace socket prostheses as the first line treatment.

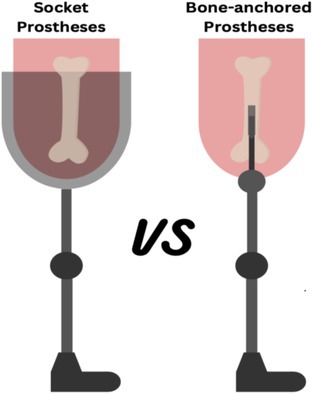

## 1 Introduction

### 1.1 Background and Context

**FIGURE 1:**
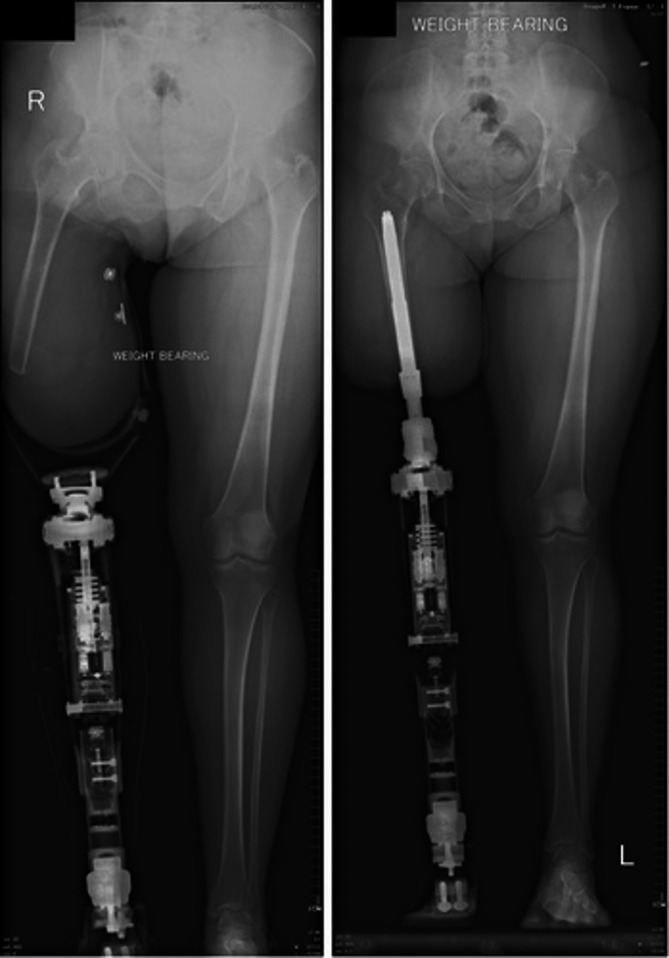
Socket prosthesis (left), Bone‐anchored prostheses (BAP) (right) [4].

Amputation is a life‐changing event that negatively impacts patients' overall quality of life (QoL) [1]. It is estimated that there are approximately 40 million amputees [2], of which 36 million (90%) are lower‐limb amputees, about 26% of whom are transfemoral amputees [3]. The current treatment for lower limb amputation is socket prosthesis (Figure 1). However, it commonly presents with socket‐related problems, impacting the patient's functionality [2, 5]. These include skin discomfort, inadequate suspension, excessive sweating, poor control, and overall low confidence [6].

Osseointegration involves the direct insertion of bone‐anchored prostheses (BAP) into the residual bone, eliminating the need for a socket prosthesis (Figure 1) [7]. The most common BAPs are the OPRA (Osseointegrated Prostheses for Rehabilitation of Amputee, Integrum AB, Sweden), ILP (Integral Leg Prostheses, ESKA Orthopedic Handels GmbH, Germany), and OPL (Osseointegrated Prosthetic Limb, Permedica, Australia). The OPRA, the only screw‐type BAP, was the first to be developed, followed by the press‐fit ILP and OPL [8]. Screw‐type implants promote bone growth around the screw threads, while press‐fit implants rely on bone growth directly into the implant surface [9]. Another difference is the bone‐skin‐implant interface: screw‐type implants aim for direct bone‐epidermis healing, while press‐fit implants use materials to limit bacterial adhesion at the tissue opening [8].

Currently, the BAP is limited to patients with socket issues, good health, and adequate stump length. Contraindications include diabetes, vascular disease, smoking, and immunosuppressive treatments due to infection risks [10]. This selective criteria exclude a large subset of amputees, limiting BAP success to specific populations. Mild infections are common but manageable, while serious complications are rare [9, 11]. Despite these risks, BAP's benefits outweigh the infection concerns [12]. Bone resorption is another complication that compromises the structural integrity of the bone‐implant interface, potentially leading to mechanical issues such as implant loosening. Although rare, it remains a significant concern [13]. For example, Atallah et al. reported three cases of aseptic loosening, underscoring the risks associated with compromised bone integrity [14].

Moreover, BAP is associated with higher overall costs, averaging €78,417 per patient compared to €54,825 for socket prostheses. A cost‐effectiveness analysis estimated an incremental cost of €83,374 per quality‐adjusted life year (QALY) gained with BAP, indicating a relatively high cost for the improvement in quality of life. However, this cost‐effectiveness improves significantly if the quality of life for socket users declines over time, suggesting that BAP may be a more viable alternative for patients experiencing socket‐related complications [15].

### 1.2 Literature Review

The latest review by Rehani et al. (2024) reported significant improvements in outcomes with BAP [6], which was supported by past reviews [11, 16, 17]. However, there are conflicting findings that suggest BAP may not significantly enhance QoL or functional outcomes for individuals already satisfied with socket prostheses [1, 5]. These reviews presented limitations such as various study designs, making it difficult to draw definitive conclusions. Additionally, inconsistent data reporting and outcome measures made conducting a meta‐analysis infeasible [6, 11, 16, 17].

### 1.3 Significance of Research

To overcome past limitations, this review focuses on a fixed outcome measure, such as Questionnaire for Persons with a Transfemoral Amputation (Q‐TFA) and 36‐Item Short Form Survey (SF‐36) for meta‐analysis. Furthermore, by only including single‐arm trials, this minimizes individual differences between populations and allows direct comparisons between participants. Lastly, by conducting a subgroup analysis on screw‐type and press‐fit BAPs, the study enhances statistical power and provides more comprehensive findings.

### 1.4 Research Question

Does BAP result in a significant improvement in Quality of Life (QoL) for transfemoral amputees compared to socket prostheses?

### 1.5 Study Aims

This review primarily aims to evaluate whether BAP significantly improves quality of life (QoL) compared to socket prostheses in transfemoral amputees. A secondary aim is to determine whether BAP leads to a significant improvement in mobility and to assess whether there is a significant difference in QoL outcomes between screw‐type and press‐fit implants.

## 2 Materials and Methods

### 2.1 Study Design

This systemic review and meta‐analysis aim to compare the quality of life (QoL) between bone‐anchored prostheses (BAP) and socket prostheses in transfemoral amputees. The review includes single‐arm trials that compare pre‐ and post‐operative outcomes. This is done in accordance with the PRISMA and Cochrane Handbook of Systematic Reviews of Interventions criteria [18]. This review was conducted at Barts Cancer Institute, Queen Mary University of London, from November 2023 to July 2024. The protocol was registered on the Prospective Register of Systematic review (PROSPERO) under the registration number CRD42024529489 (available at https://www.crd.york.ac.uk/PROSPERO/display_record.php?RecordID=529489).

### 2.2 Search Strategy

The search was conducted in the following databases: PubMed, Cochrane, EMBASE, Web of Science and Scopus, with the last search carried out in 04/05/2024. The PICO format was used to guide the search strategy. A set of search terms and keywords was applied, including: “transfemoral”, “above knee”, “lower limb”, “transfemoral amput*”, “bone‐anchored”, “osseointegrat*”, “leg‐prosthe*”, “bone‐anchored prosthesis” [MeSH], “osseointegration” [MeSH], “socket prosthe*”, and “socket‐suspended”. The search strategy across the databases utilized free words, Boolean operators (AND, OR), truncation and medical subject headings (MeSH) terms. Outcomes were not included to maximize the number of papers retrieved. The comprehensive search strategy is detailed in the Appendix S1. Additionally, the reference lists of relevant articles and gray literature were manually screened. Articles requiring institutional access were acquired through university library. Authors of papers with no access were contacted through email.

### 2.3 Inclusion and Exclusion Criteria

The eligibility criteria for this review are categorized based on the Population, Interventions, Comparisons, Outcomes, and Study design (PICOS) framework [19], and illustrated in Table 1.

**TABLE 1:** Inclusion and exclusion criteria.

	Inclusion	Exclusion
Population	Unilateral or bilateral transfemoral amputee Over 18 years old	Amputation at other level
Intervention	Post‐operative performance of any model of BAP	
Comparators	Studies including pre‐operative performance of socket users +/− non‐socket users due to socket‐related problems or wheelchair users	Studies with only non‐socket users
Outcomes	Quality of Life (QTFA, SF‐36), Mobility (6MWT) No restrictions on follow‐up time Raw data must be available as mean (± SD)	Did not report data on transfemoral separately
Study design	Single‐arm trial study that compares pre‐ and post‐operative outcomes	Other languages other than English Cross‐sectional studies or other study designs that are not single‐arm trial

Abbreviations: 6MWT, 6‐min walk test; BAP, bone‐anchored prostheses; Q‐TFA, questionnaire for persons with a transfemoral amputation; SD, standard deviation; SF‐36, 36‐item short form survey.

### 2.4 Study Selection and Data Extraction

All studies from the search were uploaded into Rayyan and EndNote for removal of duplicates. Both authors (JT and NZ) independently screened the titles and abstracts of retrieved references for the relevant studies. Then, the full text of all potentially relevant studies was extracted and further screened based on pre‐defined inclusion and exclusion criteria. Any disagreement was resolved by discussion and asked for another author (RK) if needed. The review authors recorded all reasons for exclusion through labeling on the Rayyan website. Data extracted from the included studies included the name of the author, published year, region, study design, follow‐up period, participants demographics, type of intervention, outcome measurement, and result findings. All data were extracted and synthesized using Excel and Word document (Table 2).

**TABLE 2:** Summary characteristics of included studies.

Study	Region	Study design	Follow‐up period	Number of participants, *n* (U/B)	Comparator	Type of BAP	Outcome measurement	Summary of results
Hagberg 2008	Sweden (Mölndal)	Prospective study. single‐arm trial	2 years	15 (14/1)	Socket only	OPRA	Q‐TFA (all), SF‐36 (PCS, MCS)	Significant improvement in all domains of Q‐TFA and SF‐36 (PCS). No significant improvement in SF‐36 (MCS)
Van de Meent 2013	Netherlands (Nijmegen)	Prospective case control. single‐arm trial	1 years	22 (21/1)	Socket only	ILP (referred as OIP in study)	Q‐TFA (global score, prosthesis use), 6MWT	Significant improvement in Q‐TFA (global, prosthesis use) and 6MWT
Hagberg 2014	Sweden (Gothenburg)	Prospective case control. Single‐arm trial	2 years	39 (39/0)	Socket (*n* = 33), non‐socket (*n* = 6)	OPRA	Q‐TFA (all), SF‐36 (PCS)	Significant improvement in all domains of Q‐TFA and SF‐36 (PCS)
Muderis 2016	Australia (Sydney)	Prospective cohort, single‐arm trial	21.5 months	50 (50/0)	Socket (*n* = 36), non‐socket/wheelchair users (*n* = 14)	OPL and ILP	Q‐TFA Global, SF‐36 (PCS), 6MWT	Significant improvement in Q‐TFA global, SF‐36 (PCS) and 6MWT
Muderis 2018	Australia	Prospective, case series, single‐arm trial	36.38 months	37 (37/0)	Socket and wheelchair users	OPL and ILP	Q‐TFA global, SF‐36 (PCS), 6MWT	Significant improvement in Q‐TFA global, SF‐36 (PCS), 6MWT
Atallah 2020	Netherlands (Nijmegen)	Prospective cohort, single‐arm trial	1 year	16 (15/1)	Socket (*n* = 8), non‐socket (*n* = 8)	OFI‐Y	Q‐TFA (global, prosthesis use)	Significant improvement in Q‐TFA (global, prosthesis use)
McMenemy 2020	UK	Retrospective cohort study, single‐arm trial	2 years	7 (0/7)	Socket only	OPL	SF‐36 (PCS, MCS), 6MWT	Significant improvement in SF‐36 (PCS, MCS) and 6MWT
Hagberg 2020	Sweden (Mölndal)	Prospective cohort, single‐arm trial	7 years	111 (111/0)	Socket only	OPRA	Q‐TFA (all)^§^	Significant improvement in all domains of Q‐TFA
Reif 2021	United States (New York)	Retrospective, single‐arm trial	21.1 months	18 (NR)	Socket and non‐socket	OPL	Q‐TFA (all), 6MWT	Significant improvement in all domains of Q‐TFA and 6MWT
Rojas 2021	South America (Chile)	Retrospective case series, single‐arm trial	5 years	21 (NR)	Socket only	POP	Q‐TFA (all)	Significant improvement in all domains of Q‐TFA
Sinclair 2022	United States (Salt Lake City, UT)	Prospective cohort, single‐arm trial	52 weeks	10 (10/0)	Socket only	POP	Q‐TFA (all), 6MWT	Significant improvement in all domains of Q‐TFA and 6MWT
Hagberg 2023	Sweden (Mölndal)	Prospective study, single‐arm trial	10 years	51 (45/6)	Socket (*n* = 31), non‐socket (*n* = 6)	OPRA	Q‐TFA (all), SF‐36 (PCS, MCS)	Significant improvement in all domains of Q‐TFA and SF‐36 (PCS). No significant improvement in SF‐36 (MCS)
Haidary 2023	NR	Retrospective study, Single‐arm trial	6.3 years	1 (0/1)	Socket only	OPL	Q‐TFA (global, prosthetic mobility, problem), SF‐36 (PCS, MCS), 6MWT	Significance value is not reported, but improvement is observed in Q‐TFA (Global, Prosthetic mobility, problem), SF‐36 (PCS, MCS) and 6MWT

*Note:* If data reported, the values in parentheses in the comparator column represent the number of participants, indicated as (*n*=).

Abbreviations: 6MWT, 6 min walk test; b, bilateral; BAP, bone‐anchored prostheses; ILP, integral leg prosthesis; MCS, mental component score; *n*, number of participants; NR, not reported; OFI‐Y, Gamma osseointegration femur implant; OIP, osseointegration prosthesis; OPL, osseointegrated prosthetic limb; OPRA, osseointegrated prostheses for the rehabilitation of amputees; PCS, physical component score; POP, percutaneous osseointegrated prosthesis; Q‐TFA, questionnaire for a persons with a transfemoral amputation; SF‐36, 36‐item short form health survey; U, unilateral.

The primary outcome, Quality of life (QoL), was measured with the Questionnaire for Persons with a Transfemoral Amputation (Q‐TFA) and 36‐Item Short Form Health Survey (SF‐36). The Q‐TFA is a self‐reported questionnaire that consists of four domains: Global, Prosthetic Mobility, Problem, and Prosthetic Use; each one is scored from 0 to 100 points. A higher score indicates better outcomes in Prosthetic Use, Prosthetic Mobility, and the Global domain, except for the Problem domain, where a lower score signifies better results [20]. The SF‐36 consists of two summary measures: Physical Component Summary (PCS) and Mental Component Summary (MCS) score [11, 21]. The secondary outcome, mobility, was measured with the 6‐min walk test (6MWT).

### 2.5 Methodological Quality

The ROBINS‐I (Risk of Bias in Non‐randomized Studies—of Interventions) tool assessed the risk of bias for non‐randomized controlled trials (NRCTs) based on seven domains: confounding variable, participant selection, classification of intervention, deviation from the intended intervention, missing data, measurement of outcome, and selection of reported result. The overall bias for the study is determined as either low (comparable to a randomized study), moderate (sound for a non‐randomized study, but not comparable to a randomized study), serious (study has some important problems in this domain), or critical (study is too problematic to provide any useful evidence) [22]. Publication bias was assessed by the asymmetry of the funnel plot. Publication bias is suspected to be present if there is an unequal spread of studies around the effect estimate line.

The GRADE approach (Grading of Recommendations Assessment, Development and Evaluation certainty of evidence) was used to evaluate the level of evidence using GRADEpro Guideline Development Tool software by Cochrane. This is determined by looking at factors: risk of bias, inconsistency, imprecision, indirectness, and publication bias. The GRADE has four levels of evidence: very low (true effect is likely significantly different from the estimated effect), low (true effect is possibly significantly different from the estimated effect), moderate (true effect is likely close to the estimated effect), and high (true effect is similar to the estimated effect) [23].

### 2.6 Statistical Analysis

To assess both primary and secondary outcomes, descriptive statistics were used to calculate the total number of patients and the average mean score across all included studies. Meta‐analysis was performed solely for the primary outcome measurements, Q‐TFA and SF‐36, by calculating the mean difference using an inverse variance random‐effects model with a 95% confidence interval (CI). The random‐effects model accounts for some level of heterogeneity among the intervention effects observed in different studies, with this variation reflected in the Tau^*2*^ test. Outcomes where *p* < 0.05 are considered statistically significant. Studies qualified for meta‐analysis must have sample size, mean, and standard deviation (SD) data. Review Manager (RevMan) 5.4 version software (The Cochrane Collaboration, Oxford, UK) was used to generate a forest plot. Subgroup analyses were performed to compare the difference in QoL between the press‐fit and screw‐type BAP. This was assessed with the *p*‐value, where *p* < 0.05 indicates a significant difference between subgroups. Sensitivity analyses were performed via removal of studies including non‐socket users to assess the effect on the *I* ^2^ score (measurement of heterogeneity). This was performed in studies with a high heterogeneity (*I* ^2^ > 50%). *I* ^2^ value of less than 25% indicates low heterogeneity, *I* ^2^ between 25% and 50% indicates moderate heterogeneity, and *I* ^2^ greater than 50% indicates high heterogeneity [24].

## 3 Results

### 3.1 Description of Studies

**FIGURE 2:**
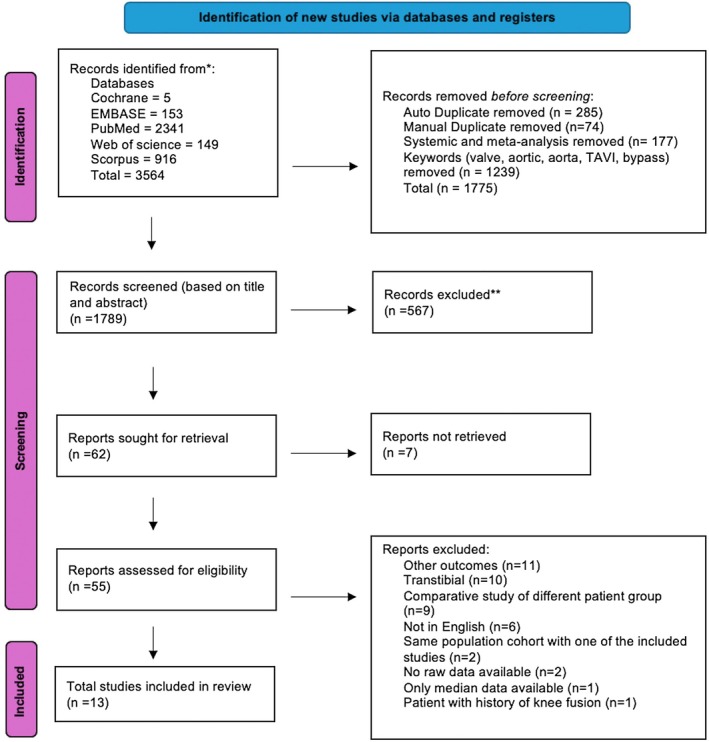
PRISMA chart flow diagram.

The screening process of this review is illustrated in the PRISMA flow diagram Figure 2. 13 NRCTs were included with a total number of 398 patients [14, 25, 26, 27, 28, 29, 30, 31, 32, 33, 34, 35, 36]. Table 3 shows the study characteristics and main findings summarized. The inclusion of only single‐arm trials is to minimize individual differences between populations and allow direct comparisons between participants. The BAP are categorized into two subgroups: screw‐type and press‐fit. The three most common BAP implants included in this review are the OPRA, ILP, and OPL. Other BAPs included are the OFI‐Y (Gamma press‐fit osseointegration femur implant, Permedica S.p.A., Italy) and the POP (Percutaneous osseointegrated prosthesis, DJO Surgical, Austin, TX), which are both press‐fit [27, 28].

**TABLE 3:** Summary of patient demographics.

Study	Number of participants, *n* (U/B)	Amputation side, *n* (R/L)	Age at implantation, years (mean ± SD/median and range)	Gender, *n* (M/F)	Cause of amputation, *n*	Years after amputation (mean ± SD/range)
Hagberg 2008	15 (14/1)	NR	44.6 (22–62)	6/9	Trauma =11 Tumor = 4	17.3 (2–33)
Van de Meent 2013	22 (21/1)	NR	46.5 ± 10.7	18/4	Trauma = 20 Tumor = 2	16.4 ± 14.8
Hagberg 2014	39 (39/0)	NR	44 ± 12.4	17/22	Trauma =23 Tumor =11 Other =5	NR
Muderis 2016	50 (50/0)	25/25	48.4 (24–73)	34/16	Trauma = 32 Blast = 3 Infection = 5 Oncology = 8 Congenital = 2	NR
Muderis 2018	37 (37/0)	NR	NR	NR	NR	NR
Atallah 2020	16 (15/1)	8/7, B (1)	50 ± 15	13/3	Trauma =11 Infection =1 Tumor = 3 Congenital =2	17 (8–28)
McMenemy 2020	7 (0/7)	NR	28 (24–33)	7/0	Blast = 7	NR
Hagberg 2020	111 (111/0)	59/52	44.6 ± 12.6	78/33	Trauma = 75 Tumor = 23 Emboli = 3 Infectio*n* = 10	11.1 ± 10.8
Reif 2021	18 (NR)	NR	49.6 ± 12	11/7	Trauma = 13 Necrotizing fasciitis =1 Infection = 2 Vascular injury = 2	7.8 ± 8.8
Rojas 2021	21 (NR)	10/11	43 (26–61)	18/3	Trauma =21	NR
Sinclair 2022	10 (10/0)	6/4	48 ± 12	10/0	Blast = 5 Motor vehicle collision = 2 Trauma = 3	9.4 ± 5.7
Hagberg 2023	51 (45/6)	NR	44 ± 12	28/23	Trauma = 33 Tumor = 12 Infection = 4 Embolus = 2	12 ± 11
Haidary 2023	1 (0/1)	B (1)	48 ± 0	0/1	Burn = 1	47^a^
	*N* = 398 (342/17) NR = 39		Mean = 46.8	*N* = 240/121 NR = 37	Total Trauma = 239 Tumor/oncology = 63 Infection = 22 Other = 38 NR = 36	Mean = 13.0

Abbreviations: B, bilateral; F, female; L, left; M, male; *n*, number of participants; *N*, total number of participants; NR, not reported; Other, (blast, embolus, congenital, necrotizing fasciitis, vascular, motor vehicle collision, burn); R, right; SD, standard deviation; U, unilateral.

^a^ Excluded from mean calculation due to outlier.

The OPRA is the only screw‐type implant and is used in 4 out of 13 studies, while the others are press‐fit implants. The OPL is the most used implant, reported in 5 out of 13 studies. The length of follow‐up ranges from 1 to 10 years. For studies that provided data at multiple follow‐ups, the latest one was selected [14, 27, 31, 34]. In the study by Hagberg, the 7‐year follow‐up data was reported instead of the 15‐year data due to the unavailability of raw data [30]. The study by Hagberg reported the longest patient follow‐up at 10 years [14]. This was a continuation study from Branemark et al. who reported on the outcomes at a 2‐year and 5‐year follow‐up [37].

Table 4 summarizes the demographics of participants included in the study. All participants in this review are transfemoral amputees, comprising 240 men and 121 women, resulting in a 2:1 ratio. The mean age at implantation was 46.8 years, and the mean time since amputation was 13 years. The mode reason for amputation is trauma. The pre‐operative comparator group included both socket users and non‐socket users. The number of participants varied between the post‐operative BAP group and the pre‐operative socket group due to the unavailability of data or patient loss during follow‐up [14, 27, 30, 34, 36]. Reasons for patient loss during follow‐up include implant loosening [26, 35], aseptic loosening [25, 29], infection [25, 29], fractures [25, 26, 29], dropouts [25], and death [25, 29].

**TABLE 4:** BAP vs. socket prostheses: Q‐TFA global score.

			Socket				BAP				*p*			
Study	Number of participants, *n* (U/B)	Follow‐up period	Global (mean ± SD)	Prosthetic (mean ± SD)	Problem (mean ± SD)	Prosthetic use (mean ± SD)	Global (mean ± SD)	Prosthetic mobility (mean ± SD)	Problem (mean ± SD)	Prosthetic use (mean ± SD)	Global	Prosthetic mobility	Problem	Prosthetic use
Hagberg 2008	18 (16/2)	2 years	37.73 (*n* = 15)	57.53 (*n* = 15)	38.07 (*n* = 15)	51.06 (*n* = 18)	72.12 (*n* = 17)	65.94 (*n* = 17)	16.53 (*n* = 17)	82.89 (*n* = 18)	0.002	0.001	0.002	0.013
Van de Meent 2013	22 (21/1)	1 year	39 ± 4.7	NR	NR	56 ± 7.9	63 ± 5.3	NR	NR	101 ± 2.4	0.001	NR	NR	< 0.001
Hagberg 2014	39 (39/0)	2 years	38 ± 18.5 (*n* = 33)	56 ± 16.2 (*n* = 33)	43 ± 19.1 (*n* = 33)	52 ± 36.7 (*n* = 39)	76 ± 17.4 (*n* = 38)	69 ± 16.1 (*n* = 38)	16 ± 11.3, (*n* = 38)	84 ± 24.2 (*n* = 39)	< 0.0001	< 0.0001	< 0.0001	< 0.0001
Muderis 2016	50 (50/0)	21.5 months	47.82 ± 17.28	NR	NR	NR	83.52 ± 18.04	NR	NR	NR	< 0.001	NR	NR	NR
Muderis 2018	37 (37/0)	36.36 months	45.27 ± 3.96	NR	NR	NR	84.86 ± 3.39	NR	NR	NR	< 0.0001	NR	NR	NR
Atallah 2020	16 (15/1)	1 year	31 ± 18 (*n* = 8)	NR	NR	31 ± 41 (*n* = 16)	79 ± 10 (*n* = 8)	NR	NR	93 ± 12 (*n* = 16)	< 0.01	NR	NR	< 0.01
Hagberg 2020	111 (111/0)	7 years	NR	NR	NR	NR	74 ± 20.6 (*n* = 54)	67 ± 17.8 (*n* = 54)	17 ± 10.8 (*n* = 54)	85 ± 25 (*n* = 54)	< 0.001	< 0.001	< 0.001	< 0.001
Reif 2021	18 (NR)	1 year	17.6	45.55	51	49.77	77.08	66.29	20.17	75.23	Y	Y	Y	Y
Rojas 2021	17 (NR)	5 years	35.77 ± 21.2	46.89 ± 20	50.38 ± 21.7	54.56 ± 30.7	74.45 ± 15.3	79.08 ± 13.6	22.54 ± 22.9	89.35 ± 13.4	< 0.0001	< 0.0001	0.0001	0.0001
Sinclair 2022	10 (10/0)	52 weeks	62 ± 21	64 ± 24	25 ± 14	78 ± 32	92 ± 11 (*n* = 8)	81 ± 21 (*n* = 8)	3 ± 2 (*n* = 8)	96 ± 5 (*n* = 8)	< 0.001	0.03	< 0.001	0.02
Hagberg 2023	51 (45/6)	10 years	37.7 ± 19.3 (*n* = 42)	52.5 ± 20.4 (*n* = 42)	43.9 ± 18.7 (*n* = 42)	46.7 ± 36.7 (*n* = 51)	74 ± 24.4 (*n* = 35)	65.8 ± 18.7 (*n* = 35)	16.3 ± 12.6 (*n* = 35)	80.6 ± 26.1 (*n* = 36)	< 0.001	< 0.001	< 0.001	< 0.001
Haidary 2023	1 (0/1)	6.3 years	66.67	56.67	19.58	NR	66.67	52.78	10.00	NR	^a^	^a^	^a^	^a^
	*N* = 382		Total = 41.57 *N* = 245	Total = 54.94 *N* = 128	Total = 37.74 *N* = 128	Total = 49.68 *N* = 183	Total = 76.63 *N* = 297	Total = 66.65 *N* = 180	Total = 15.15 *N* = 180	Total = 84.50 *N* = 220				

*Note:* For studies where the number of patients in the outcome measures differs from the initial number of patients included, this is indicated in parentheses as (*n*=). Not all studies reported SD.

Abbreviations: B, bilateral; BAP, bone‐anchored prostheses; *n*, number of participants; *N*, total number of participants; NR, not reported; Q‐TFA, questionnaire for persons with a transfemoral amputation; SD, standard deviation; U, unilateral; Y, *p*‐value not reported but qualitatively stated as statistically significant.

^a^ *p*‐value undetermined as only one participant.

### 3.2 Risk of Bias

**FIGURE 3:**
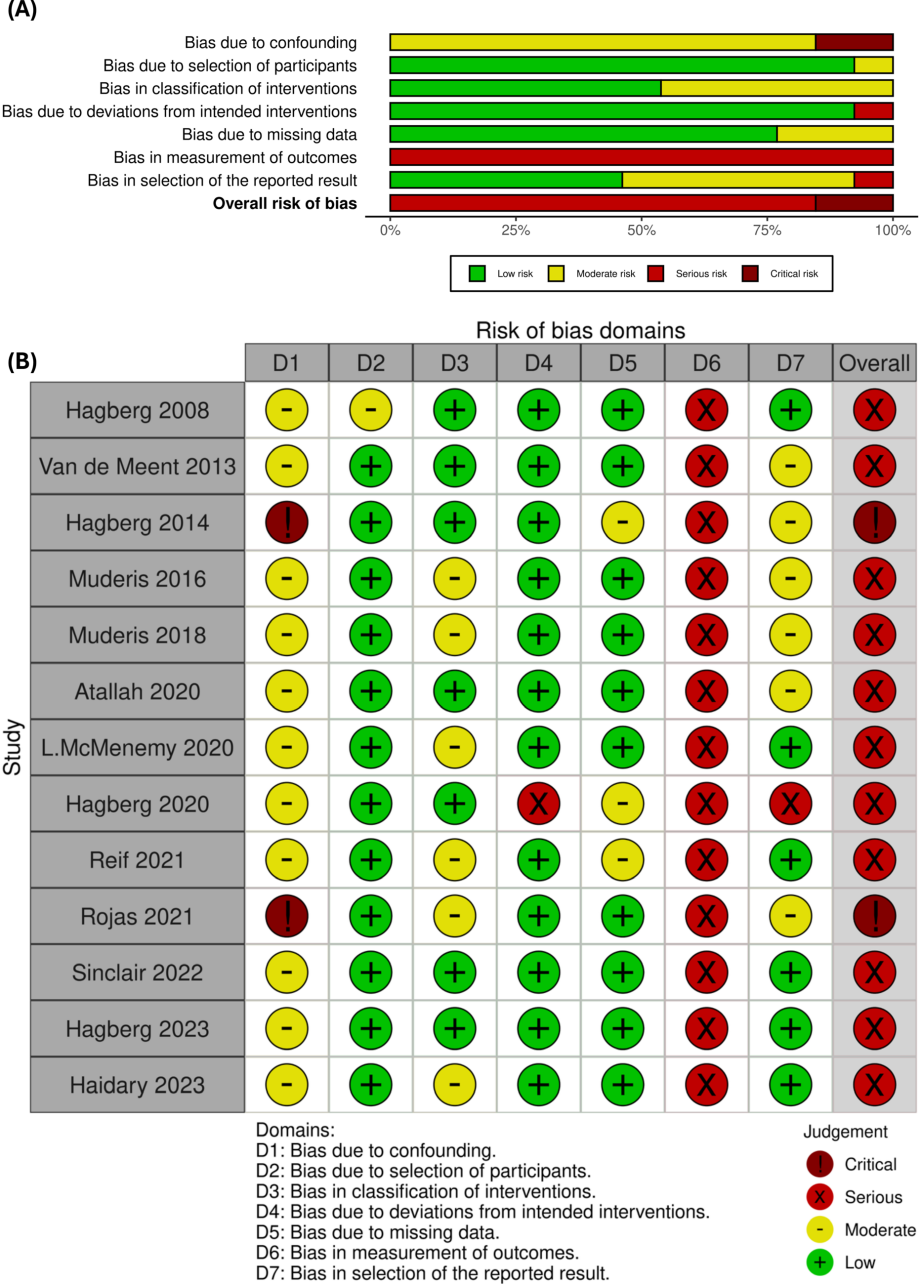
The ROBINS‐I risk of bias assessment of included studies. (A) Risk of bias graph for included studies; (B) Risk of bias summary for included studies. ROBINS‐I; Risk of Bias in Non‐randomized Studies—of Interventions.

The risk of bias was assessed with ROBINS‐I and illustrated in Figure 3. Thirteen studies were included in this meta‐analysis with 11 studies rated as “serious” and 2 studies as “critical”. This is mainly due to the lack of blinding of intervention and the subjective nature of outcome measurements, such as the Q‐TFA and SF‐36 questionnaires. Publication bias was assessed with funnel plots, which is detailed in the Appendix S2. This was not assessed in SF‐36 (MCS) as meta‐analysis was not conducted. Overall, asymmetry is present in all funnel plots, suggesting publication bias, but the assessment is unreliable due to an insufficient number of studies, which is below the Cochrane recommended minimum of 10 studies.

### 3.3 Descriptive Statistics for Quality of Life (QoL)

Quality of life measured with Q‐TFA was reported in 12 studies, with each of the 4 domains (Global, Prosthetic Mobility, Problem, and Prosthetic Use) analyzed separately (Table 5). The Q‐TFA Global score was reported in 12 studies. The mean Q‐TFA Global score in the socket group (249 participants) to the BAP group (298 participants) increased from 41.69 to 76.49. The 11 out of 12 studies reported significant improvement when comparing the socket group to the BAP group [14, 27, 28, 29, 30, 31, 32, 33, 34, 35, 36]. The Q‐TFA Prosthetic Mobility score was reported in eight studies. The mean Q‐TFA Prosthetic Mobility score in the socket group (136 participants) to the BAP group (185 participants) increased from 54.02 to 69.59. The 6 out of 8 studies reported significant improvement when comparing the socket group to the BAP group [14, 28, 29, 30, 34, 36]. One study by Sinclair et al. showed no significant difference between the socket group and the BAP group [27]. The Q‐TFA Problem score was reported in eight studies. The mean Q‐TFA Problem score in the socket group (136 participants) to the BAP group (185 participants) decreased from 38.70 to 15.37. The 7 out of 8 studies reported significant improvement when comparing the socket group to the BAP group [14, 27, 28, 29, 30, 34, 36]. The mean Q‐TFA Prosthetic Use score was reported in nine studies. The mean Q‐TFA Prosthetic Use score in the socket group (188 participants) to the BAP group (224 participants) increased from 53.67 to 88.39. All 9 studies reported significant improvement when comparing the socket group to the BAP group [14, 27, 28, 29, 30, 31, 34, 35, 36].

**TABLE 5:** BAP vs. socket prostheses: SF‐36.

			Socket		BAP		*p*	
Study	Number of participants, *n* (U/B)	Follow up period	PCS (mean ± SD)	MCS (mean ± SD)	PCS (mean ± SD)	MCS (mean ± SD)	PCS	MCS
Hagberg 2008	15 (14/1)	2 years	31	55	44	50	> 0.05	> 0.05
Hagberg 2014	39 (39/0)	2 years	32.1 ± 9.1	NR	40.5 ± 9.8	NR	< 0.0001	NR
Muderis 2016	50 (50/0)	21.5 months	37.09 ± 9.54 (*n* = 46)	NR	47.29 ± 9.33 (*n* = 49)	NR	< 0.001	NR
Muderis 2018	37 (37/0)	36.38 months	36.97 ± 1.51	NR	49.00 ± 1.71	NR	< 0.0001	NR
McMenemy 2020	7 (7/0)	2 years	34.65	41.55	54.5	58.19	0.018	0.018
Hagberg 2023	51 (45/6)	10 years	33 ± 8 (*n* = 50)	53 ± 12 (*n* = 50)	39 ± 10 (*n* = 37)	52 ± 13 (*n* = 37)	0.001	0.3
Haidary 2023	1 (0/1)	6.3 years	34.82	63.08	46.92	31.11	^a^	^a^
	*N* = 203		Total = 34.25 *N* = 195	Total = 53.16 *N* = 73	Total = 53.54 *N* = 185	Total = 47.83 *N* = 60		

*Note:* For studies where the number of patients in the outcome measures differs from the initial number of patients included, this is indicated in parentheses as (*n*=). Not all studies reported SD.

Abbreviations: B, bilateral; BAP, bone‐anchored prostheses; MCS, mental component score; *n*, number of participants; *N*, total number of participants; NR, not reported; PCS, physical component score; SD, standard deviation; SF‐36, Short Form 36 Health Survey Questionnaire; U, unilateral.

^a^ *p*‐value undetermined as only 1 participant.

Quality of life measured with SF‐36 was studied in 7 out of 13 studies, with the two summary measures PCS and MCS analyzed separately (Table 6). The SF‐36 PCS score was analyzed in seven studies. The mean SF‐36 PCS score in the socket group (195 participants) to the BAP group (185 participants) increased from 34.25 to 53.54. Five studies reported significant improvement when comparing the socket group to the BAP group [14, 25, 32, 33, 34]. One study showed no significant improvement between the socket group and the BAP group [36]. The SF‐36 MCS score was analyzed in four studies. The mean SF‐36 MCS score in the socket group (73 participants) to the BAP group (60 participants) decreased from 53.16 to 47.83. Only one study by McMenemy et al. reported a significant increase in the SF‐36 MCS score [25]. Two studies reported no significant difference between the socket group and the BAP group [14, 36]. The *p*‐value for Haidary et al. study could not be determined due to the inclusion of only a single participant [26].

**TABLE 6:** BAP vs. socket prostheses: 6MWT(m).

Study	Number of participants, *n* (U/B)	Follow up period	Socket, *m* (mean ± SD)	BAP, *m* (mean ± SD)	*p*
Van de Meent 2013	22 (21/1)	1 year	321 ± 28	423 ± 21	0.002
Muderis 2016	36 (36/0)^a^	NR	281 ± 93	419 ± 133	< 0.001
Muderis 2018	37 (37/0)	NR	286.25 ± 21.63	412.72 ± 23.69	< 0.0001
McMenemy 2020	7 (7/0)	2 years	248	402	0.018
Reif 2021	18 (NR)	NR	564.6	955	< 0.05
Sinclair 2022	10 (10/0)*	1 year	481 ± 146 (*n* = 10)	584 ± 96 (*n* = 8)	< 0.001
Haidary 2023	1 (0/1)	6.3 years	0	437.5	^b^
	*N* = 131		Total = 311.69 *N* = 131	Total = 519.03 *N* = 129	

*Note:* For studies where the number of patients in the outcome measures differs from the initial number of patients included, this is indicated in parentheses as (*n*=). Not all studies reported SD.

Abbreviations: 6MWT, 6‐min walk test; B, bilateral; BAP, bone‐anchored prostheses; *m*, meter; *n*, number of participants; *N*, total number of participants; NR, not reported; SD, standard deviation; U, unilateral.

^a^ Only socket users data included as no pre‐op data for wheelchair users.

^b^ *p*‐value undetermined as only 1 participant.

### 3.4 Meta‐Analysis for Quality of Life (QoL)

**FIGURE 4:**
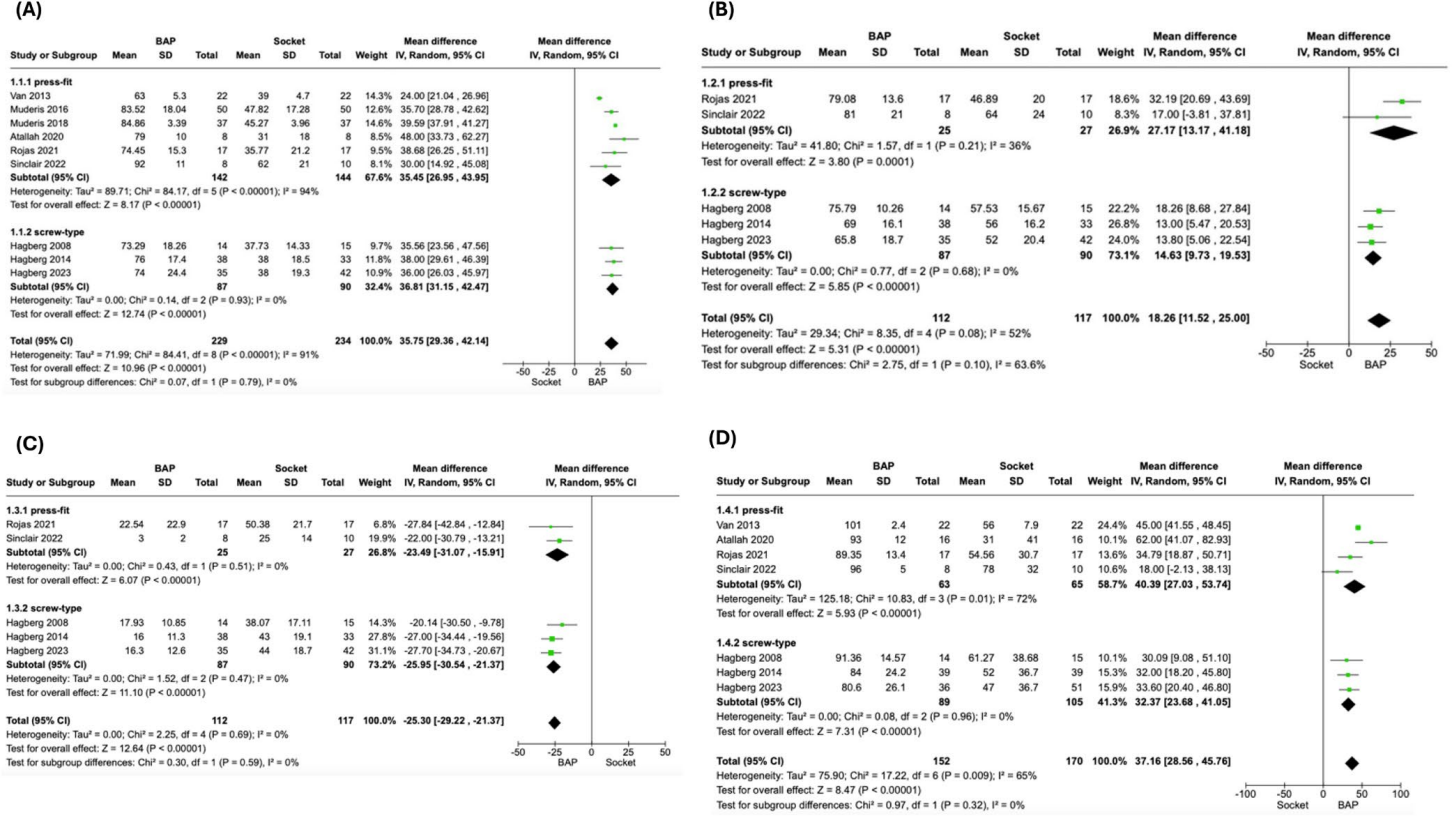
Forest Plot comparing Q‐TFA scores in BAP vs. Socket Prostheses in transfemoral amputee. (A) Q‐TFA Global Score; (B) Q‐TFA Prosthetic Mobility Score; (C) Q‐TFA Problem Score; (D) Q‐TFA Prosthetic Use Score. Q‐TFA, Questionnaire for a Persons with a Transfemoral Amputation; BAP, bone‐anchored prostheses; SD, standard deviation; IV, inverse variance; CI, confidence interval; df, degrees of freedom.

For Q‐TFA Global Score, nine studies reported a mean difference of 35.75 (95% CI: 29.36–42.14; *p* < 0.00001) (Figure 4A) [14, 27, 28, 31, 32, 33, 34, 35, 36]. For Q‐TFA Prosthetic Mobility score, five studies reported a mean difference of 18.26 (95% CI: 11.52–25.00, *p* < 0.00001) (Figure 4B) [14, 27, 28, 34, 36]. For Q‐TFA Problem score, five studies reported a mean difference of −25.30 (95% CI: −29.22 to −21.37, *p* < 0.00001) (Figure 4C) [14, 27, 28, 34, 36]. For Q‐TFA Prosthetic Use score, seven studies reported a mean difference of 37.16 (95% CI: 28.56–45.76, *p* < 0.00001) (Figure 4D) [14, 27, 28, 31, 34, 35, 36]. The four Q‐TFA domains (Global, Prosthetic Mobility, Problem and Prosthetic Use) showed no significant difference between press‐fit and screw‐type subgroups. The subgroup difference in Global, Prosthetic Mobility, Problem, and Prosthetic Use domain was *p* = 0.79, *p* = 0.10, *p* = 0.59, *p* = 0.32 respectively (Figure 4A–D).

**FIGURE 5:**
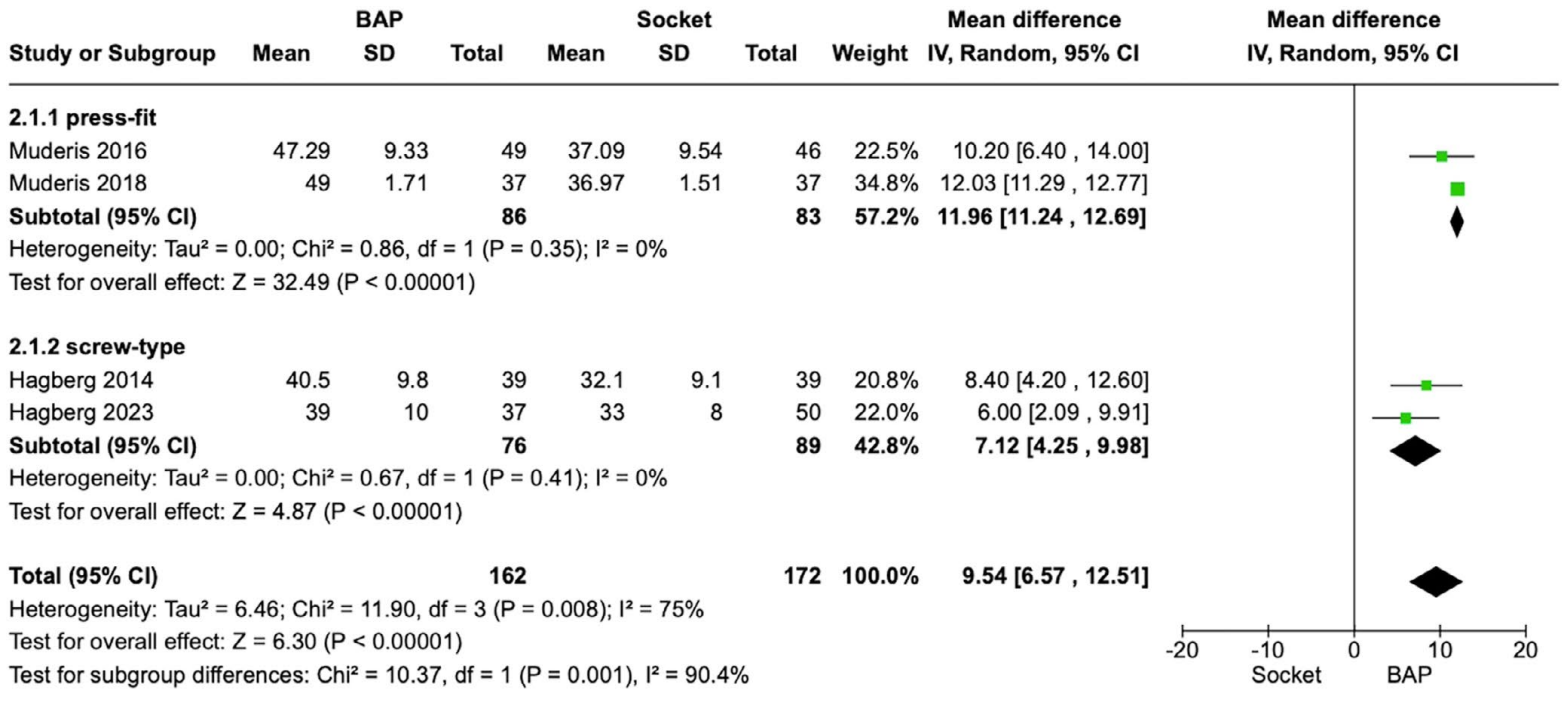
Forest Plot comparing SF‐36 (PCS) in BAP vs. Socket Prostheses in transfemoral amputee. SF‐36, 36‐Item Short Form Health Survey; PCS, Physical Component Score; BAP, bone‐anchored prostheses; SD, standard deviation; IV, inverse variance; CI, confidence interval; df, degrees of freedom.

For SF‐36 PCS, four studies reported a mean difference of 9.54 (95% CI: 6.57–12.51, *p* < 0.00001) (Figure 5) [14, 32, 33, 34]. The subgroup difference was *p* = 0.001 indicating a significantly better result in the press‐fit subgroup. Meta‐analysis was not performed for the MCS domain due to an insufficient number of studies reporting SD data.

### 3.5 Descriptive Statistics for Mobility

The 6MWT was measured in 7 out of 13 papers (Table 7). The mean 6MWT score in the socket group (131 participants) to the BAP group (129 participants) increased from 311.69 to 519.03. Six studies reported a significant difference between the socket group and the BAP group [25, 27, 29, 32, 33, 35].

**TABLE 7:** Summary of findings.

Comparison of quality of life in BAP vs socket prostheses in transfemoral amputee: A systemic review and meta‐analysis
Patient: Transfemoral amputee that are pre‐operatively using socket prosthesis or not using prosthesis/using ambulatory device (due to socket related problems) Intervention: Bone‐anchored prostheses (BAP). This includes separate analyses of press‐fit and screw‐type implant system Comparison: Socket prostheses or non‐prosthesis users Study type: Systematic Review and Meta‐analysis (single‐arm trials)

Outcome	Number of studies	Number of participants (BAP: socket)	Absolute effect (95% CI)	Certainty of evidence (GRADE)	Comment
Q‐TFA: global score Follow up: 1–10 years	Overall				
	9	229:234	MD 35.75 higher (29.36 higher to 42.14 higher)	⨁◯◯◯ Verylow^a^, ^b^, ^c^, ^d^	Results are statistically significant overall with no significant difference between subgroups. However, results are based on a small number of NRTCs with high risk of bias, affecting precision of results. There is also significant heterogeneity overall, but none in subgroup analyses.
	Press‐fit				
	6	142:144	MD 35.45 higher (26.95 higher to 43.95 higher)	⨁◯◯◯ Very low^a^, ^b^, ^c^, ^d^	
	Screw‐type				
	3	87:90	MD 36.81 higher (31.15 higher to 42.47 higher)	⨁⨁◯◯ Low^a^, ^c^, ^d^	
Q‐TFA: prosthetic mobility score Follow up: 1–10 years	Overall				
	5	112:117	MD 18.26 higher (11.52 higher to 25 higher)	⨁◯◯◯ Very low^a^, ^c^, ^d^, ^e^	Results are statistically significant overall with no significant difference between subgroups. However, results are based on a small number of NRTCs with high risk of bias, affecting precision of results. There is also moderate heterogeneity present overall and between subgroups
	Press‐fit				
	2	25:27	MD 27.17 higher (13.17 higher to 41.18 higher)	⨁⨁◯◯ Low^a^, ^c^, ^d^	
	Screw‐type				
	3	87:90	MD 14.63 higher (9.73 higher to 19.53 higher)	⨁⨁◯◯ Low^a^, ^c^, ^d^	
Q‐TFA: problem score Follow up: 1–10 years	Overall				
	5	112:117	MD 25.3 lower (29.22 lower to 21.37 lower)	⨁⨁◯◯ Low^a^, ^c^, ^d^	Results are statistically significant overall with no significant difference between subgroups. However, results are based on a small number of NRTCs with high risk of bias, affecting precision of results. There is no heterogeneity present overall and between subgroups
	Press‐fit				
	2	25:27	MD 23.49 lower (31.07 lower to 15.91 lower)	⨁⨁◯◯ Low^a^, ^c^, ^d^	
	Screw‐type				
	3	87:90	MD 25.95 lower (30.54 lower to 21.37 lower)	⨁⨁◯◯ Low^a^, ^c^, ^d^	
Q‐TFA: prosthesis use score Follow up: 1–10 years	Overall				
	7	152:170	MD 37.16 higher (28.56 higher to 45.76 higher)	⨁◯◯◯ Very low^a^, ^c^, ^d^, ^e^	Results are statistically significant overall with no significant difference between subgroups. However, results are based on a small number of NRTCs with high risk of bias, affecting precision of results. There is moderate heterogeneity present overall, but no heterogeneity between subgroups
	Press‐fit				
	4	63:65	MD 40.39 higher (27.03 higher to 53.74 higher)	⨁◯◯◯ Very low^a^, ^b^, ^c^, ^d^	
	Screw‐type				
	3	89:105	MD 32.37 higher (23.68 higher to 41.05 higher)	⨁⨁◯◯ Low^a^, ^c^, ^d^	
SF‐36 physical component score (PCS) Follow up: 21.5 months to 10 years	Overall				
	4	162:172	MD 9.54 higher (6.57 higher to 12.51 higher)	⨁◯◯◯ Very low^a^, ^c^, ^d^, ^e^	Results are statistically significant overall with no significant difference between subgroups. However, results are based on a low number of NRTCs with high risk of bias, affecting precision of results. There is no heterogeneity present overall and between subgroups
	Press‐fit				
	2	86:83	MD 11.96 higher (11.24 higher to 12.69 higher)	⨁⨁◯◯ Low^a^, ^c^, ^d^	
	Screw‐type				
	2	76:89	MD 7.12 higher (4.25 higher to 9.98 higher)	⨁⨁◯◯ Low^a^, ^c^, ^d^	
SF‐36 physical component score (MCS) Follow up: 21.5 months to 10 years	4	60:73	MD 5.33 lower Mean socket group score is 53.16 compared to BAP mean score 47.83.	⨁⨁◯◯ Low^a^	No meta‐analysis for this outcome as insufficient number of studies. The small mean difference, 5.33, suggests no significant difference. Low number of NRCTs with high risk of bias and small sample size lower the certainty of evidence.
6 min walk test (6MWT) Follow up: 1–3 years	7	67:69	MD 207.34 higher Mean BAP group score is 519.03 compared to socket mean score 311.69	⨁⨁◯◯ Low^a^	No meta‐analysis for this outcome. The large mean difference, 207.34, suggests significant difference. Low number of NRCTs with high risk of bias and small sample size lower the certainty of evidence.

*Note:* Relative risk can't be calculated due to design of study as before‐after event.

Abbreviations: BAP, bone‐anchored prostheses; CI, confidence interval; GRADE, grading of recommendations assessment, development and evaluation certainty of evidence; Q‐TFA, questionnaire for a persons with a transfemoral amputation; MD, mean difference; NRCTs, non‐randomized controlled trials; SF‐36, 36‐item short form health survey; PCS, physical component score; MCS, mental component score; 6MWT, 6 min walk test.

^a^ Most information is from studies at high risk of bias. This is generally due to lack of blinding of intervention and subjective outcome measurement.

^b^ *I* ^2^ value (> 75%) indicates significantly high heterogeneity. The presence of < 10 studies and small sample sizes contributes significantly to this heterogeneity.

^c^ Imprecision is largely due to few numbers of studies included and small sample size (< 400 is below Cochrane recommended threshold).

^d^ Publication bias is inconclusive due to < 10 studies included and small sample size (Appendix S2).

^e^ *I* ^2^ test (50%–75%) indicates moderate heterogeneity. The presence of < 10 studies and small sample size contribute to this heterogeneity.

### 3.6 Sensitivity Analyses

**FIGURE 6:**
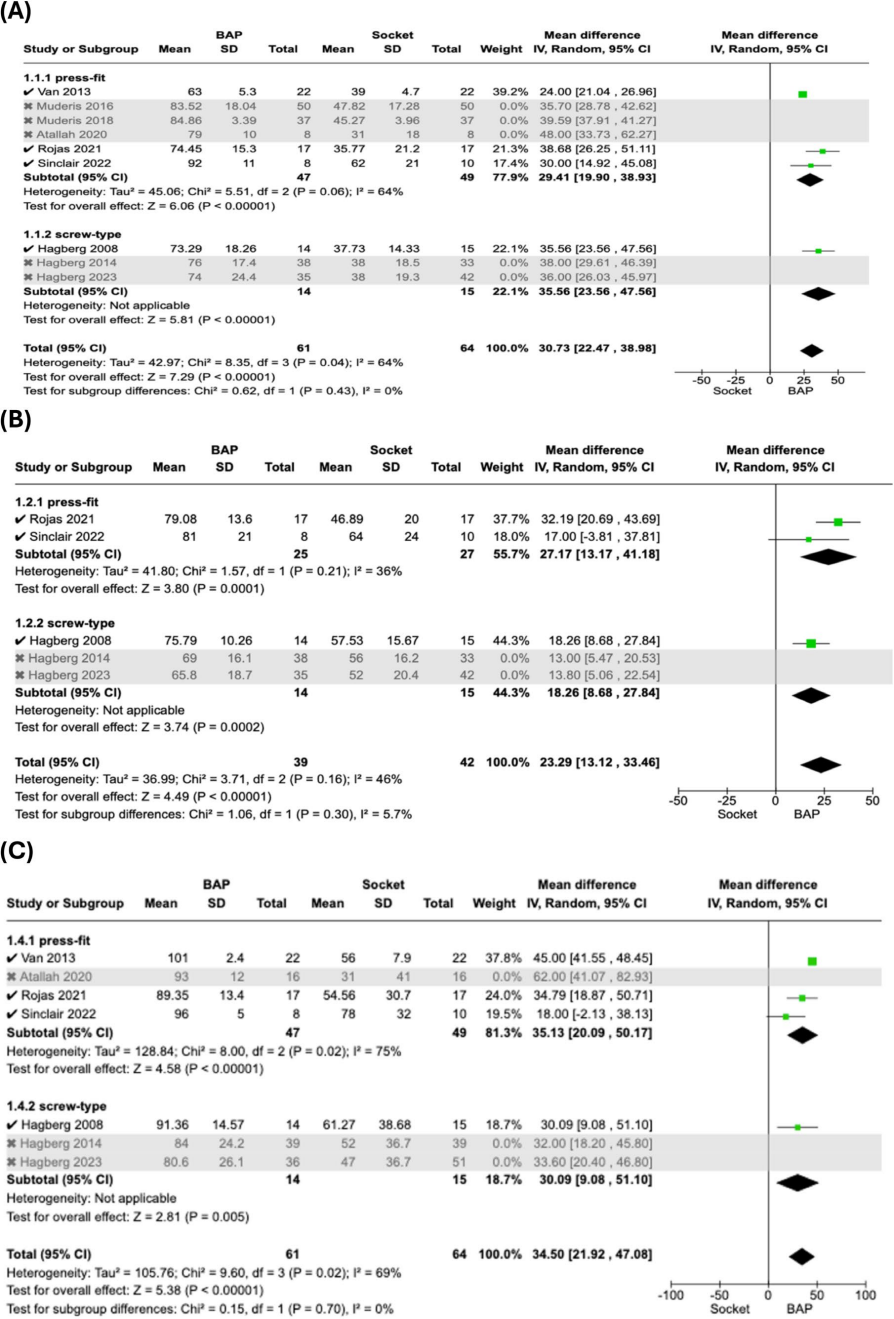
Sensitivity analyses comparing Q‐TFA scores in BAP vs. Socket Prostheses in transfemoral amputee. This was carried out by removing studies including non‐socket users. (A) Q‐TFA Global Score; (B) Q‐TFA Prosthetic Mobility Score; (C) Q‐TFA Prosthetic Use Score. Q‐TFA, Questionnaire for a Persons with a Transfemoral Amputation; BAP, bone‐anchored prostheses; SD, standard deviation; IV, inverse variance; CI, confidence interval; df, degrees of freedom.

For Q‐TFA Global score, the *I* ^2^ score was reduced from 91% to 64% (Figure 6A) [14, 31, 32, 33, 34]. For Prosthetic Mobility score, the *I* ^2^ score was reduced from 52% to 46% (Figure 6B) [14, 34]. For Problem score, as the *I* ^2^ score was 0%, sensitivity analysis was not performed. For Prosthetic Use score, the *I* ^2^ score increased from 65% to 69% (Figure 6C) [14, 31, 34]. For SF‐36 PCS, as all the studies included non‐socket users, sensitivity analysis was not performed.

### 3.7 Summary of Findings

The Summary of Findings table, shown in Table 7, summarizes the key outcome findings of this review, including effect estimates and evaluates the quality of evidence for each outcome using the GRADE approach.

## 4 Discussion

### 4.1 Aim

To the best of our knowledge, this is the first meta‐analysis to systematically compare the quality of life between socket prostheses and BAP, based exclusively on the inclusion of single‐arm trials. Unlike prior reviews that included mixed study designs, our use of single‐arm trials allows for within‐subject comparisons, offering a more precise estimate of individual benefit. The findings showed that BAP significantly improves quality of life and mobility compared to socket prostheses, as measured by Q‐TFA, SF‐36, and 6MWT. Additionally, this is the first study to conduct subgroup analyses, which overall revealed no significant difference in quality of life between press‐fit and screw‐type BAP implants.

### 4.2 Q‐TFA

The review showed that BAP had significantly better outcomes across all four Q‐TFA domains, with the Q‐TFA Global score being the most measured. The Q‐TFA Global score also showed the most improvement with a mean increase of 35.06. This reflects substantial functional and psychosocial benefits in everyday life with the BAP.

Sensitivity analyses (*I* ^2^ = 91%–64%) indicated minimal impact with the inclusion of non‐socket users. Thus, it indicates that these improvements are robust across non‐socket and socket users. This is supported by Rehani et al. who showed improved Q‐TFA Global scores at 1–15 years follow‐up periods [6]. Moreover, this review is consistent with previous studies, demonstrating a strong positive correlation between Q‐TFA Prosthetic Mobility and SF‐36 PCS, with both showing significant results (*p* < 0.00001) [11].

The significant findings in the Q‐TFA Problem domain are due to the direct insertion of the BAP, eliminating socket‐tissue discomfort [5]. The heterogeneity of 0% suggests consistency among the included studies. However, the Q‐TFA Problem domain is designed for socket prostheses and does not account for BAP complications like infection and implant loosening [20, 37]. Despite that, Branemark et al. included these factors and still showed significant differences (*p* < 0.0001), supporting this review findings [37]. This suggests that such complications are rare and that the benefits of BAP outweigh the associated risks.

Besides that, Q‐TFA Prosthetic Use showed the second greatest improvement with a mean increase of 34.83. This highlights the enhanced prosthetic integration and user confidence with the BAP. Sensitivity analyses had minimal impact on the Q‐TFA Prosthetic Use score, suggesting variation across all studies. Notably, heterogeneity is only found in the press‐fit subgroup. This could be due to the use of different types of press‐fit BAP implants: POP, OFI‐Y, and ILP, each with a slightly different design, potentially influencing result [27, 28, 31, 35]. Furthermore, Atallah et al. reported the highest improvement with the OFI‐Y implant, a derivative of OPL [31]. This aligns with literature where OPL is the preferred and most used implant, as reported in 5 out of 13 studies. The OPL is favored for its single‐stage implantation procedure and shorter rehabilitation time [7, 8]. Hagberg et al. found significant improvements in Q‐TFA Prosthetic Use scores at 10 years (*p* < 0.0001), but not at 15 years (*p* = 0.094), likely due to mechanical complications being apparent after 10 years [30]. The increase in mechanical complications is positively correlated with activity level. This is suspected to affect individuals after 10 years, thus reducing prosthesis use [30]. Therefore, longer follow‐up studies are needed to strengthen the findings of this review.

### 4.3 SF‐36

The significant SF‐36 (PCS) findings align with literature showing BAP offers better range of motion and control than socket prostheses [5, 8]. Though Rojas et al. did not report PCS scores, significant *p*‐values in the four domains of the PCS: Physical Functioning (PF), Role functioning from a Physical Perspective (RP), Bodily Pain (BP) and General Health (GH) suggest likely significance [28]. The strong correlation between PCS, Q‐TFA Prosthetic Mobility, and Prosthetic Use scores, as noted by Hagberg et al., strengthens these findings [20].

The SF‐36 (MCS) was the least reported with the smallest sample size, showing no significant difference due to a small mean difference of 5.33 and inconsistent results [26]. Only the study by McMenemy et al. reported a significant improvement in the MCS score, which may be attributed to the lower mean age of participants and who were combat amputees. This may contribute to more positive mental health outcomes and greater adaptability to BAP, whereas older amputees tend to report lower MCS scores and may be less receptive to such interventions [25].

Our findings revealed that there is a greater focus on physical health‐related QoL, with more studies reporting PCS scores and less on MCS scores [33, 34, 36]. This discrepancy may arise because amputation significantly impacts functional levels, which is more detectable in PCS scores, and the use of a prosthesis can positively impact the physical scores. However, the non‐significant changes in MCS scores may be due to the challenges individuals face in adapting to new prostheses or psychological distress related to the amputation itself. These factors are not directly linked to prosthesis use and thus show a less drastic change. This is supported by Rehani et al., which states that improvement in QoL does not often translate to improvement in the mental health of BAP users [6].

### 4.4 Subgroup Analyses

Our subgroup analysis is the first to compare QoL outcomes across different BAP implant types. The absence of significant differences in all the Q‐TFA domains between press‐fit and screw‐type implants suggests that both designs offer comparable functional outcomes. Notably, the press‐fit subgroup demonstrated significantly better SF‐36 (PCS) scores, indicating a potential physical advantage.

Subgroup analyses in all the Q‐TFA domains revealed no significant difference. This suggests that both types are equally effective, despite the preference for OPL [7, 8]. Contrastingly, only SF‐36 (PCS) revealed a significantly better outcome with press‐fit BAP. However, high heterogeneity (*I* ^2^ = 90.4%) in this analysis signals variability across implant types and warrants cautious interpretation. Additionally, with 13 low‐quality NRCTs with high risk of bias, this affects the reliability of this finding and should be interpreted with caution. Nevertheless, based on the insignificant difference in the Q‐TFA score, it is concluded that there is no significant difference in QoL between press‐fit and screw‐type BAP.

### 4.5 6MWT

Our findings indicate a substantial increase in walking distance following BAP implantation, with mean 6MWT scores rising from 311.69 m (socket) to 519.03 m (BAP). This nearly 70% improvement highlights the significant functional benefit of BAP in real‐world mobility. For mobility, Sinclair et al. found no significant difference in 6MWT at 5 weeks (*p* = 0.64) but significant improvement at 12 weeks (*p* = 0.001) [27], consistent with the 2–3 month weight‐bearing period for BAP implants [7]. Additionally, one of the included studies showed no significant difference between individuals with and without infection (*p* = 0.29), suggesting it minimally affects function and reinforces its clinical use [35]. Moreover, mobility positively correlates with QoL, emphasizing rehabilitation's role in restoring mobility [38]. The improved 6MWT and significant gains in our analysis of Q‐TFA Prosthetic Mobility, Q‐TFA Prosthetic Use, and SF‐36 (PCS) scores (*p* < 0.00001) further support BAP implants' effectiveness in enhancing amputee function.

### 4.6 Limitations

While this meta‐analysis offers important insights, several limitations must be acknowledged. Firstly, it consists of NRCTs with a high risk of bias, mainly due to unblinded intervention and subjective outcome measurements. Besides that, while heterogeneity in the follow‐up period could affect results, there were no significant differences found between studies. This is supported by Hagberg et al. and Branemark et al. who found no significant differences between 5‐ and 10‐year follow‐ups [14], and 2‐ and 5‐year follow‐ups respectively [37]. However, follow‐up variation should still be considered.

This review also included too few studies, below Cochrane's threshold, and had a small sample size of 398 participants, leading to unreliable heterogeneity estimates and potentially reducing the statistical power of the study. This emphasizes the need for larger, high quality RCTs to validate our findings. Furthermore, many studies had inconsistent pre‐ and post‐op patient numbers due to follow‐up losses, and missing data wasn't handled with statistical analyses. Moreover, some studies were by the same author, introducing selective reporting and publication bias. There were four studies published by Hagberg et al. [14, 30, 34, 36] and 2 studies published by Muderis et al. [32, 33] The studies by Hagberg et al. represent a longitudinal series with overlapping cohorts but report on different time points and patient subsets. It is recognized that the partial overlap of participants may have inflated effect sizes or introduced bias.

This review also included both socket and non‐socket users, introducing confounding and affecting result precision. While sensitivity analyses excluded non‐prosthesis users, too few studies remained to form firm conclusions. Lastly, many studies excluded patients with diabetes, vascular injury, or smokers—contraindications for the BAP procedure [7]. Only Rojas et al. included a diabetic patient, but no specific data was available [28]. This limits the generalizability of results, as it does not represent the broader amputee population with comorbidities. Hence, future studies should include a broader population.

## 5 Conclusions

This review highlights the potential of BAP to significantly improve both quality of life and mobility in transfemoral amputees. These benefits were observed consistently across implant types, reinforcing the clinical value of BAP as a viable alternative to socket prostheses. However, significant limitations—such as high risk of bias in existing studies, small sample sizes, exclusion of patients with comorbidities, and the relatively high cost of BAP—currently restrict its use to individuals with socket‐related complications. As such, while BAP represents a promising alternative, it cannot yet be universally recommended as a first‐line intervention. Further high‐quality RCTs involving larger and more diverse patient populations are essential to support broader clinical adoption.

## Author Contributions

**Janice Tan:** conceptualization, methodology, writing – original draft, visualization, writing – review and editing, data curation. **Nafisa Zilani:** conceptualization, data curation, methodology, writing – review and editing. **Rezaul Karim:** writing – review and editing, methodology, data curation, conceptualization. **Bijendra Patel:** supervision, writing – review and editing.

## Disclosure

All authors have met the authorship criteria according to the latest guidelines of the International Committee of Medical Journal Editors, and all authors are in agreement with the manuscript.

## Ethics Statement

The authors have nothing to report.

## Consent

The authors have nothing to report.

## Conflicts of Interest

The authors declare no conflicts of interest.

## Supporting information


**Data S1.** Supporting Information.

**Data S2.** Supporting Information.

## Data Availability

The data that support the findings of this study are available from the corresponding author upon reasonable request.
