# Supplementary material for: Comparison of Quality of Life in Transfemoral Amputee Using Bone‐Anchored Prostheses vs. Socket Prostheses: A Systemic Review and Meta‐Analysis
Source: Orthop Surg. 2025 Jul 18;17(8):2234–54. doi: 10.1111/os.70086 (PMC12318688; doi:10.1111/os.70086)
Supplement: Supplementary file 1 — Data S1. Supporting Information. [file OS-17-2234-s002.docx]

**Table S1.** PubMed Advanced Search

| Search | Query (Search Terms) | Number of Results |
| --- | --- | --- |
| (#1) Patient | Transfemoral OR “above knee” OR “lower limb” OR “lower extremity” OR “transfemoral amput*” OR “lower amputation” | 111,809 |
| (#2) Intervention | “Bone-anchored” OR “bone anchored” OR osseointegrat* OR “leg prosthe*” OR “leg-prosthe*” OR “press-fit” OR “press fit” OR “screw-type” OR “screw type” OR OPRA OR ILP OR EEP OR OPL OR Bone-anchored prosthesis[MeSH] OR Osseointegration[MeSH] OR Prosthesis Implantation / methods[MeSH] | 84,487 |
| (#3) Outcomes | "Socket prosthe*" OR "socket-suspended" OR “socket suspen*” OR "transfemoral prosthe*" OR "socket-suspension" | 63,400 |
| Total | #1 AND #2 AND #3 | 2341 |

**Table S2.** Cochrane Advanced Search

| Search | Query (Search Terms) | Number of Results |
| --- | --- | --- |
| (#1) Patient | Transfemoral OR (above NEXT knee) OR (lower NEXT limb) OR (lower NEXT extremity) OR (transfemoral NEXT amput*) OR (lower NEXT amputation) OR MeSH descriptor:[Lower Extremity] explode all trees OR MeSH descriptor:[Amputation, Surgical] explode all trees | 46,760 |
| (#2) Intervention | (Bone-anchored) OR (bone NEXT anchored) OR osseointegrat* OR (leg NEXT prosthe*) OR (leg-prosthe*) OR (press-fit) OR (press NEXT fit) OR (screw-type) OR (screw NEXT type) OR OPRA OR ILP OR EEP OR OPL OR MeSH descriptor:[Osseointegration] explode all trees OR OR MeSH descriptor:[Prosthesis implantation] explode all trees | 12,948 |
| (#3) Outcomes | "Socket prosthe*" OR "socket-suspended" OR “socket suspen*” OR "transfemoral prosthe*" OR "socket-suspension" | 20 |
| Total | #1 AND #2 AND #3 | 5 |

**Table S3.** EMBASE Advanced Search

| Search | Query (Search Terms) | Number of Results |
| --- | --- | --- |
| (#1) Patient | Transfemoral OR ‘above knee’ OR ‘lower limb’ OR ‘lower extremity’ OR ‘transfemoral amput*’ OR ‘lower amputation’ OR ‘above knee prosthesis’/exp OR ‘above knee amputation’/exp | 172, 235 |
| (#2) Intervention | ‘Bone-anchored’ OR ‘bone anchored’ OR osseointegrat* OR ‘leg prosthe*’ OR ‘leg-prosthe*’ OR ‘press-fit’ OR ‘press fit’ OR ‘screw-type’ OR ‘screw type’ OR OPRA OR ILP OR EEP OR OPL OR ‘osseointegrated implant’/ exp OR ‘osseointegration’/exp | 27,937 |
| (#3) Outcomes | ‘Socket prosthe*’ OR ‘socket-suspended’ OR ‘socket suspen*’ OR ‘transfemoral prosthe*’ OR ‘socket-suspension’ | 442 |
| Total | #1 AND #2 AND #3 | 153 |

**Table S4.** Web of Science Advanced Search

| Search | Query (Search Terms) | Number of Results |
| --- | --- | --- |
| (#1) Patient | Transfemoral OR “above knee” OR “lower limb” OR “lower extremity” OR “transfemoral amput*” OR “lower amputation” | 169,620 |
| (#2) Intervention | “Bone-anchored” OR “bone anchored” OR osseointegrat* OR “leg prosthe*” OR “leg-prosthe*” OR “press-fit” OR “press fit” OR “screw-type” OR “screw type” OR OPRA OR ILP OR EEP OR OPL OR Bone-anchored prosthesis[MeSH] OR Osseointegration[MeSH] OR Prosthesis Implantation / methods[MeSH] | 46,061 |
| (#3) Outcomes | "Socket prosthe*" OR "socket-suspended" OR “socket suspen*” OR "transfemoral prosthe*" OR "socket-suspension" | 730 |
| Total | #1 AND #2 AND #3 | 149 |

**Table S5.** Scopus Advanced Search

| Search | Query (Search Terms) | Number of Results |
| --- | --- | --- |
| (#1) Patient | Transfemoral OR “above knee” OR “lower limb” OR “lower extremity” OR “transfemoral amput*” OR “lower amputation” | 198,665 |
| (#2) Intervention | “Bone-anchored” OR “bone anchored” OR osseointegrat* OR “leg prosthe*” OR “leg-prosthe*” OR “press-fit” OR “press fit” OR “screw-type” OR “screw type” OR OPRA OR ILP OR EEP OR OPL | 48,821 |
| (#3) Outcomes | "Socket prosthe*" OR "socket-suspended" OR “socket suspen*” OR "transfemoral prosthe*" OR "socket-suspension" | 26,640 |
| Total | #1 AND #2 AND #3 | 916 |
