# Supplementary material for: Comparison of Quality of Life in Transfemoral Amputee Using Bone‐Anchored Prostheses vs. Socket Prostheses: A Systemic Review and Meta‐Analysis
Source: Orthop Surg. 2025 Jul 18;17(8):2234–54. doi: 10.1111/os.70086 (PMC12318688; doi:10.1111/os.70086)
Supplement: Supplementary file 2 — Data S2. Supporting Information. [file OS-17-2234-s001.docx]

**A)** Q-TFA Global Score **B)** Q-TFA Prosthetic Mobility Score


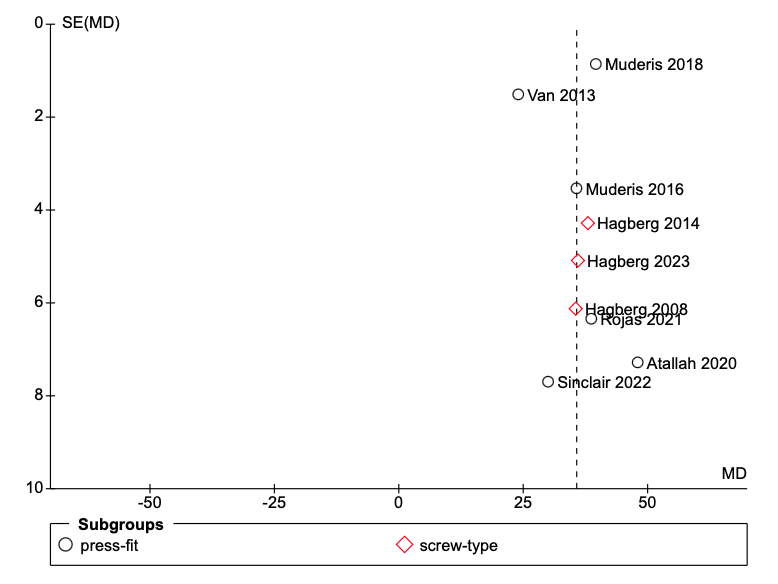

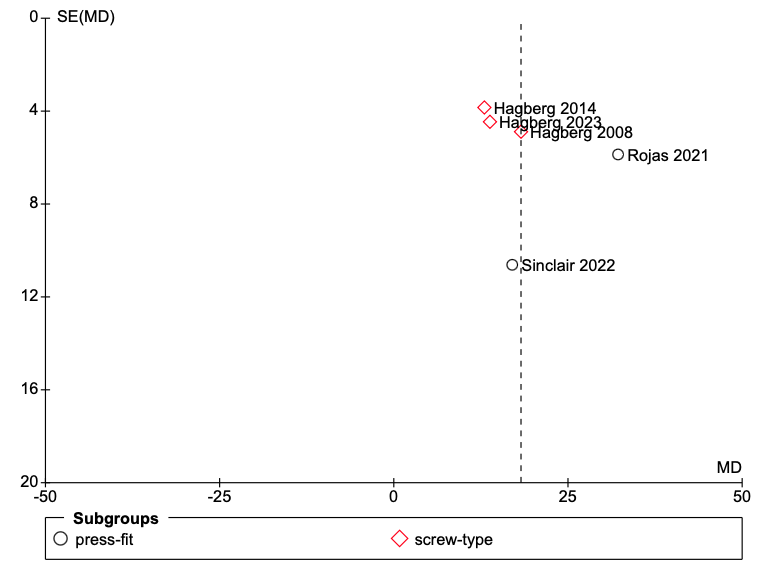


**C)** Q-TFA Problem Score **D**) Q-TFA Prosthetic Use Score


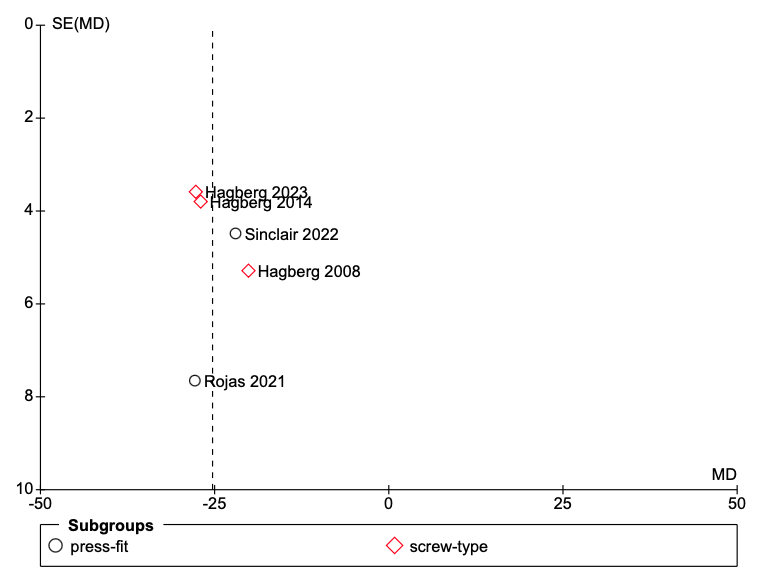

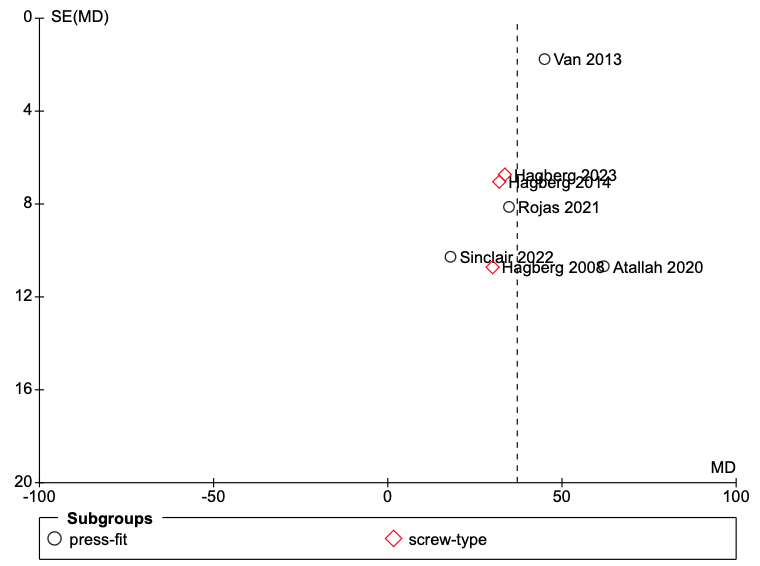


**E)** SF-36 (PCS)


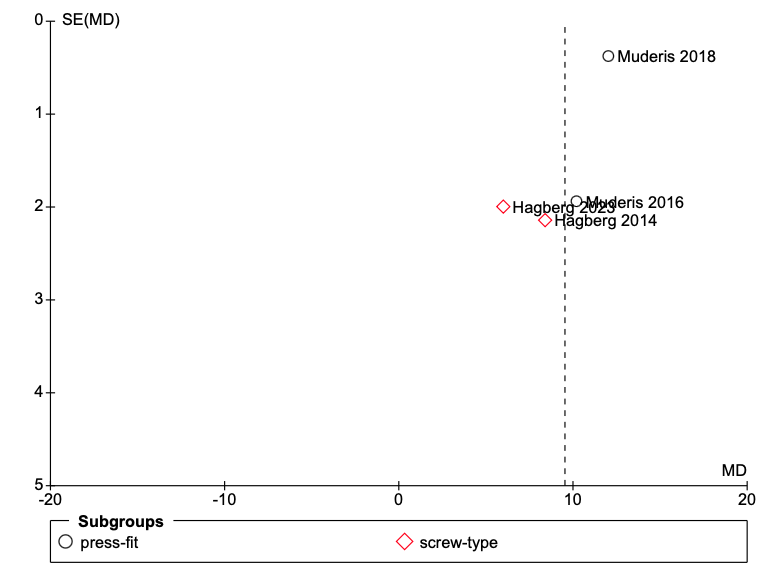


**Funnel plot of outcome measurements:** **A)** Q-TFA (Global Score) **B)** Q-TFA (Prosthetic Mobility Score) **C)** Q-TFA (Problem Score) **D)** Q-TFA (Prosthetic Use) **E)** SF-36 Physical Component Score (PCS)
